# Mental and behavioral disorders and sickness-absenteeism among federal civil servants

**DOI:** 10.47626/1679-4435-2022-800

**Published:** 2023-02-13

**Authors:** Priscila Oliveira de Miranda, Suleima Pedroza Vasconcelos

**Affiliations:** 1 Programa de Pós Graduação em Saúde Coletiva, Universidade Federal do Acre, Rio Branco, AC, Brazil

**Keywords:** mental disorders, absenteeism, sick leave, occupational health, government employees, transtornos mentais, absenteísmo, licença médica, saúde do trabalhador, servidores públicos

## Abstract

**Introduction:**

Mental disorders have been responsible for increasing sickness absenteeism, and are associated with long-term disabilities, resulting in reduced productivity and quality of life for workers.

**Objectives:**

To describe the profile of sickness absenteeism due to mental and behavioral disorders among federal civil servants in the executive branch in the state of Acre between 2013 and 2018.

**Methods:**

In this descriptive time series analysis with a quantitative design, sick leaves for mental and behavioral disorders approved by clinics of the Integrated Subsystem for Civil Servant Health Care of Acre were investigated.

**Results:**

Mental and behavioral disorders were the second main cause of absences during the study period, leading to more than 19,000 lost workdays. The prevalence of these leaves ranged from 0.81% in 2013 to 2.42% in 2018. Sick leaves due to mental disorders were granted mainly to female employees aged > 41 years for a period of 6-15 days. The most frequent diagnoses were depressive episodes, followed by other anxious disorders.

**Conclusions:**

Sickness absenteeism due to mental and behavioral disorders increased during the study period. These results reveal an urgent need for health promotion programs and prevention policies for these disorders in this population, as well as for further research to assess the impact of work conditions and the organization of work processes on the mental health of federal civil servants.

## Abstract (pt)

**Introdução:**

Os transtornos mentais têm sido responsáveis por números crescentes de absenteísmo-doença e estão associados a incapacidades de longa duração, acarretando a redução da produtividade e qualidade de vida dos trabalhadores.

**Objetivos:**

Descrever o perfil do absenteísmo-doença ocasionado por transtornos mentais e comportamentais em servidores públicos federais do Poder Executivo no estado do Acre no período de 2013 a 2018.

**Métodos:**

Tratou-se de um estudo descritivo de série temporal com abordagem quantitativa que avaliou as licenças para tratamento de saúde por transtornos mentais e comportamentais, concedidas através da perícia médica em uma das unidades do Subsistema Integrado de Atenção à Saúde do Servidor do estado do Acre.

**Resultados:**

Os transtornos mentais e comportamentais representaram a segunda principal causa de afastamentos entre os servidores durante os anos estudados, gerando mais de 19 mil dias de trabalho perdidos. A prevalência desses afastamentos variou de 0,81% no ano de 2013 a 2,42% no ano de 2018. As licenças foram concedidas principalmente a servidores do sexo feminino, com idade superior a 41 anos e com tempo de afastamento entre 6 a 15 dias. Os diagnósticos mais frequentes foram episódios depressivos, sendo seguidos por outros transtornos ansiosos.

**Conclusões:**

O absenteísmo-doença ocasionado pelos transtornos mentais e comportamentais aumentou no período do estudo. Tais resultados indicam a necessidade urgente de políticas de promoção à saúde e prevenção desses agravos a essa população e de estudos que avaliem o impacto das condições e a organização dos processos de trabalho na saúde mental dessa população de servidores.

## INTRODUCTION

Mental and behavioral disorders are “clinically significant conditions characterized by changes in thinking and mood (emotions) or by behaviors associated with personal distress and/or functional deterioration”.^1^ Given these characteristics, the relationship between mental disorders and work has gained worrying proportions worldwide, as mental disorders have been responsible for increasing sickness absenteeism and are associated with long-term disabilities, leading to reduced productivity and lower quality of life among employees.^2-4^

According to the Brazilian Ministry of Health, mental and behavioral disorders can be considered a work-related disease.^5^ Studies show that the occurrence or worsening of these disorders is related to work environments with high demand, low control, low social support, dissatisfaction, and organizational injustice.^6,7^

In this context, civil servants, as well as other classes of workers, are also susceptible to disorders or their aggravation due to work, especially with the changes that have taken place since the 1990s, including new management and work organization requiring increased productivity, goal setting, staff reductions, hiring practices that involve precarious and outsourced contracts, flexibilization of rights and remuneration, time control, and other factors that can increase the psychosocial risk of physical and mental health problems.^8^

In Brazil, studies about the profile of civil servants on sick leave point to mental and behavioral disorders as one of the main causes of sickness absenteeism.^9-11^ These results may be consequences of the negative effects of these new work environments on employee health.

Sickness absenteeism, ie, missing work due to illness, is a complex phenomenon with a multifactorial etiology, usually resulting from an interaction between personal and work issues. It can be considered an indicator of working conditions, quality of life, and working relationships.^12^ The early recognition and identification of the causes and characteristics of sickness absenteeism due to mental and behavioral disorders can contribute to the organization of services and the development of indicators to help formulate policies to promote health and prevent mental disorders among federal civil servants. Thus, this study aimed to describe the profile of sickness absenteeism due to mental and behavioral disorders among federal civil servants in the state of Acre, Brazil.

## METHODS

This was a descriptive time series analysis with a quantitative approach. The sample consisted of active federal civil servants in the executive branch in Acre who were examined at a *Subsistema Integrado de Atenção à Saúde do Servidor* (Integrated Public Servant Health Care Subsystem [SIASS]) clinic and granted a leave for health treatment between January 1, 2013 and December 31, 2018. Civil servants undergo official examination when presenting a doctor’s prescription for more than 5 days of sick leave or if the prescription does not meet the criteria for an administrative leave. Medical or dental leaves of up to 5 consecutive days are exempt from official review, provided that the total number of days is < 15 in the 12-month period from the start date of the first leave and that the prescription clearly identifies both the employee and the physician, including the physician’s registration number with the state board of medicine, the name of the disease or aggravation of a disease, whether coded or not, and the probable number of missed days.

The two SIASS units in Acre are associated with the Universidade Federal do Acre and the Ministry of Health and serve the other participating government agencies. We selected 2013 as the initial point of the study because it was the beginning of effective functioning for the SIASS units.

During the study period, 3,767 health care leaves were granted for all causes to a total of 3,197 employees, resulting in 102,384 lost workdays. All civil servants whose officially reviewed sick leaves due to diseases described in chapter V (Mental and Behavioral Disorders [F00-F99]) of the International Statistical Classification of Diseases and Related Health Problems - 10th Revision (ICD-10) between 2013 and 2018 were included in this study. Employees of agencies not linked to the Sistema Integrado de Administração de Pessoal - Módulo Saúde (Integrated Personnel Administration System - Health Module [SIAPE-SAÚDE]) were excluded due to administrative secrecy.

The aggregated, anonymous data referring to sick leaves in the SIAPE-SAÚDE database were obtained from the management of the SIASS units and stored in a Microsoft Excel spreadsheet. SIAPE-SAÚDE, a system for recording work health and safety data for civil servants in agencies of the federal executive branch, keeps aggregated data that can be extracted to determine management indicators. This system, which is available online through SIAPEnet, is protected by secrecy and security protocols and can only be accessed by authorized civil servants.^13^

The data, which refer to the total number of civil servants by year and by sex, were collected from the personnel files from the Ministério do Planejamento, Orçamento, e Gestão (Ministry of Planning, Budget, and Management) page. The reference month of each year for data collection was December. The data were organized and analyzed using Microsoft Excel and IBM SPSS Statistics 20.

Sick leaves, the primary variable of this study, were evaluated according to year, sex, age group, ICD-10 diagnosis, and leave length. Descriptive analyses of absolute and relative frequencies of sick leaves were performed according to these variables. The annual prevalence of sickness absenteeism due to mental disorders between 2013 and 2018 was calculated with the following formula:


Prevalence of sickness absenteeism=number of employees on leave for mental health treatment per
year/total number of employees(×100)

The chi-square test was used to compare nominal variables, and when its assumptions were violated, Fisher’s exact test was used, considering p ≤ 0.05 as significant.

This study was approved by the management of both of the involved SIASS units and the Universidade Federal do Acre Ethics Committee for Research Involving Human Beings, (13404219.1.0000.5010).

## RESULTS

Between 2013 and 2018, 3,767 sick leaves were granted to federal civil servants of the executive branch in Acre. A total of 513 (14%) of these leaves were motivated by mental and behavioral disorders, coded as F in the ICD-10. Aggravations of these disorders were the second leading cause of absence among civil servants during the study period, second only to diseases of the musculoskeletal system and connective tissue (17%). In addition, mental and behavioral disorders were the main factors involved in longer leaves, responsible for more than 19,000 lost workdays, ie, 19% of all missed days during the study period. This was followed by musculoskeletal diseases (18%) and injuries, poisoning, and consequences of external causes (11%).

**Figure 1:**
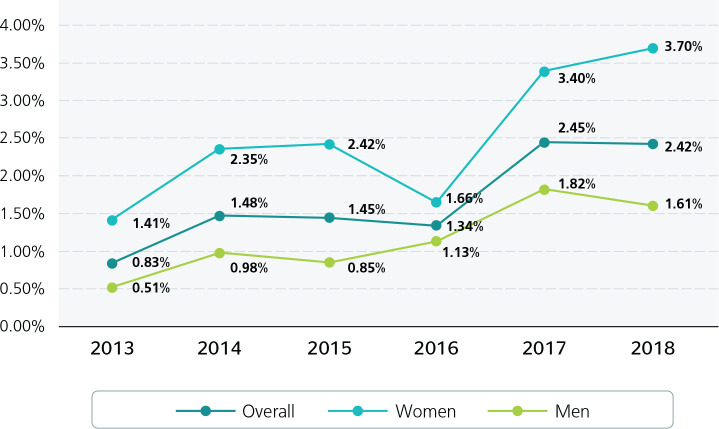
Historical series of the prevalence of sickness absenteeism due to mental and behavioral disorders among federal executive branch employees in Acre, Brazil from 2013 to 2018, overall and by sex.

The prevalence of leaves due to mental and behavioral disorders among federal civil servants grew from 0.81% in 2013 to 2.42% in 2018, ie, this type of sick leave tripled during the 6 years of the study (Figure 1).

Sick leaves due to mental disorders were granted mainly to female employees (56%) over 41 years of age (53%) and lasted between 6 and 15 days (42%). The most frequent diagnoses were depressive episodes (ICD-10 F32: 25.9%), followed by other anxiety disorders (ICD-10 F41: 20.9%). Depressive episodes (ICD-10 F32) were responsible for the most lost workdays (20.5%), followed by bipolar affective disorder (ICD-10 F31: 20.3%) (Table 1).

**Table 1:** Distribution of federal executive branch employees in Acre, Brazil on leave due to mental and behavioral disorders and days of leave according to ICD-10 diagnosis between 2013 and 2018

ICD-10 Diagnostics		Employees on leave		Days on leave	
		n	%	n	%
F32	Depressive episodes	103	25.9	3.960	20.5
F41	Other anxiety disorders	83	20.9	2.375	12.3
F31	Bipolar affective disorder	58	14.6	3.911	20.3
F33	Recurrent depressive disorder	44	11.1	2.433	12.6
F43	Reactions to severe stress and adjustment disorders	38	9.6	1.560	8.1
F19	Mental and behavioral disorders due to multiple drug use and use of other psychoactive substances	14	3.5	1.793	9.3
F10	Mental and behavioral disorders due to alcohol use	10	2.5	379	2.0
F42	Obsessive-compulsive disorder	10	2.5	354	1.8
F14	Mental and behavioral disorders due to cocaine use	8	2.0	483	2.5
F20	Schizophrenia	8	2.0	971	5.0
F29	Unspecified non-organic psychosis	7	1.8	499	2.6
F34	Persistent mood [affective] disorders	2	0.5	15	0.1
F39	Unspecified mood disorder [affective]	2	0.5	12	0.1
F40	Phobic-anxious disorders	2	0.5	17	0.1
F01	Vascular dementia	1	0.3	60	0.3
F04	Organic amnestic syndrome not induced by alcohol or other psychoactive substances	1	0.3	120	0.6
F06	Other mental disorders due to brain injury and dysfunction and physical illness	1	0.3	10	0.1
F12	Mental and behavioral disorders due to cannabinoid use	1	0.3	30	0.2
F25	Schizoaffective disorder	1	0.3	90	0.5
F45	Somatoform disorders	1	0.3	13	0.1
F71	Moderate mental retardation	1	0.3	180	0.9
F92	Mixed behavioral and emotional disorders	1	0.3	15	0.1
	Total	397	100.0	19.280	100.0

ICD-10 = International Statistical Classification of Diseases and Related Health Problems - 10th Revision.

Between 2013 and 2018, the difference between the number of leaves due to mental and behavioral disorders (513) and the number of employees on repeated leave for the same condition (397) indicates the recidivism rate. The two diagnostic subgroups responsible for the most recurrent absences were mental and behavioral disorders due to the use of psychoactive substances and mood (affective) disorders, which averaged 1.6 and 1.3 leaves per employee, respectively. Considering all-cause absences, the greatest recurrence of sick leave was due to mental and behavioral disorders, followed by the musculoskeletal and connective tissue subgroup.

As shown in Table 2, the most frequent mental disorders among women were mood (affective) disorders (64.9%), while among men they were neurotic, stress-related, and somatoform disorders (37.3%). Significant differences were observed between the sexes for mental and behavioral disorders due to psychoactive substance use (F10-F19) and for mood (affective) disorders (F30-F39), with F10-F19 being higher among men (p < 0.05) and F30-F39 being higher among women (p < 0.05).

**Table 2:** Sickness absenteeism due to mental and behavioral disorders among federal executive branch employees in Acre, Brazil from 2013 to 2018 according to sex and ICD-10 subgroups

ICD-10 F - Subgroup		Women		Men		p-value
		n	%	n	%	
F10-F19	Mental and behavioral disorders due to psychoactive substance use	1	0.4	32	18.9	0.000^*^
F20-F29	Schizophrenia, schizotypal disorders, and delusional disorders	5	2.2	11	6.5	0.057^*^
F30-F39	Mood (affective) disorders	148	64.9	62	36.7	0.000^*^
F40-F48	Neurotic, stress-related, and somatoform disorders	70	30.7	63	37.3	0.206^*^
Other groups (F00-F09, F50-F59, F60-F69, F70-F79, F80-F89, F90-F98, F99)		4	1.8	1	0.6	0.399^†^
	Total	228	100.0	169	100.0	

* Chi-square test.

† Fisher’s exact test.

ICD-10 = International Statistical Classification of Diseases and Related Health Problems - 10th Revision.

In addition to lost workdays, mental and behavioral disorders impacted other aspects of work. Between 2013 and 2018, the work capacity of 7 civil servants was assessed due to supervisor recommendation, the job description of 2 was adjusted, 13 were reassigned to other locations, and 7 were retired for disability due to mental and behavioral disorders.

## DISCUSSION

During the 6 years of the study, mental and behavioral disorders were the main cause of sick leaves among federal civil servants in the executive branch in Acre, Brazil, leading to more than 19,000 lost workdays. This result corroborates other findings that these disorders negatively impact quality of life and work capacity, as well as that affected workers miss 3 times more workdays over a 12-month period than people without these disorders.^4,14,15^

Mental and behavioral disorders were also the second leading cause (14%) of sick leave. This result agrees with other national studies that list mental and behavioral disorders as a main cause of absenteeism among civil servants in the three branches of government.^8,11,16,17^

The increased prevalence of leaves due to mental and behavioral disorders among civil servants between 2013 and 2018 reflects the growth in cases of potentially disabling mental disorders in the general population, a situation that the World Health Organization has predicted to be a public health trend.^18^ According to Instituto Nacional do Seguro Social (National Social Security Institute) data, among formally employed workers, there was a 9.23% reduction in the amount of sick pay granted for mental and behavioral disorders between 2016 and 2017. However, even with this small variation, mental and behavioral disorders remained the third leading ICD-10-listed cause of lost workdays between 2015 and 2017, behind only musculoskeletal and connective tissue diseases and injuries, poisoning, and consequences of external causes.^19^

On the contrary, there was a considerable increase in the prevalence of leaves due to mental and behavioral disorders in the study population between 2016 and 2017. One hypothesis for this increase is the economic crisis that began in mid-2014 and was accompanied and intensified by a national political crisis, culminating in the 2016 presidential impeachment. This context may have had a negative impact on the mental health of these workers, since, due to economic crisis and political instability, civil servants, mainly federal ones, suffered a lack of resources, increased duties due cutbacks in outsourced and commissioned positions, and discontinuity due to political changes.^20,21^

The results show recurrent absences due to mental and behavioral disorders, a fact that is highlighted by the higher number of sick leaves than employees who took them. These results confirm other findings that previous sick leaves can increase the risk of new leaves due to recurrent episodes, as well as early retirement due to disability.^22,23^ Multiple absences and difficulty returning to work were reported by Nielsen et al.,^24^ who, when monitoring 644 Danish workers who went on sick leave for mental and behavioral disorders over the course of 1 year, found that almost 25% of the workers had no expectations of returning to work when questioned at the beginning of the leave, 22% reported that they had previously left for the same reason, and after 1 year of absence, 12.7% still had not returned to work.

The subgroups with mood (affective) disorders and mental and behavioral disorders due to the use of psychoactive substances had the highest mean number of sick leaves per employee, reflecting the damage to the mental and physical health of psychoactive substances users and the functional impairment associated with depression, leading to recurrent and prolonged sickness absenteeism.^11,17^

The study also revealed significant sex differences in mental and behavioral disorders. Psychoactive substance use was higher among men (18.9%) than women (0.4%), whereas mood disorders were higher among women (64.9%) than men (36.7%). Such results have also been observed in studies conducted with federal and state civil servants from other regions of the country.^11,25^

The most frequent mental conditions were depressive episodes (ICD-10 F32: 25.9%), followed by other anxiety disorders (ICD-10 F41: 20.9%). Other national and international studies have also found depression and anxiety disorders to be the main causes of absenteeism among mental and behavioral disorders.^15,26,27^ The World Health Organization estimates indicate that, globally, more than 300 million people suffer from depression (4.4% of the world’s population), while anxiety-related disorders affect 264 million people (3.9%). In Brazil, these estimates are higher: depression and anxiety disorders affect 5.8 and 9.3% of the population, respectively.^18^

The majority of those who went on sick leave were women (56%). According to the World Health Organization,^26^ Schlindwein & Morais,^25^ and Fernandes et al.,^28^ women are considered vulnerable to mental disorders. This seems to be influenced by a combination of biological, psychosocial, and cultural factors that include physical and emotional conditions, the implications of the work-family interface, burnout, devaluation, violence, and better self-perceived health, ie, they tend to use health services more frequently than men.^16,29^

According to Dias et al.,^8^ considering the life cycle, older workers are more likely to take sick leaves. This was observed in the present study, since most of the sick leaves due to mental and behavioral disorders were granted to employees over 41 years of age. In addition to the decline in work capacity, another hypothesis that may better explain this phenomenon is that older workers may be under the effect of cumulative exposure to various occupational risk factors and, consequently, feelings of malaise accumulated over the years in the organizational environment, which could increase the risk of mental illness.^16^ Added to this situation is the increased prevalence of chronic diseases with advancing age. Other authors have observed that one or more comorbidity is positively associated with mental disorders.^30^

Considering the natural history of mental and behavioral disorders, some sociocultural factors among federal civil servants in Acre apparently exert some influence on their occurrence, such as feelings of not being belonging, poor adaptation to state characteristics, distance from the employee’s hometown and, in some cases, from family and established social networks. During the 6 years of this study, such factors could have been reflected in the 13 employees who were reassigned to other locations. This hypothesis was also reported in a study of federal civil servants from Tocantins, another state in northern Brazil that is similar to Acre regarding civil service.^10^

According to the International Labor Organization,^31^ prevention is key to reducing the disease burden, proving more effective and less expensive than treatment and rehabilitation. Thus, in addition to concern for worker health, it should also be recognized that these absences inconvenience institutions, since they have an economic impact, generating public expense. They compromise the performance of services, which can have a direct impact on the entire population. They can also cause burnout, job dissatisfaction, and personnel conflicts, in addition to other organizational problems.^28^

Moreover, sick leaves and other institutional adjustments to minimize the disabilities of civil servants affected by mental and behavioral disorders (eg, adapting job descriptions, transfers, work capacity assessments) have been shown to be palliative and incapable of resolving the situation, indicating a problem that seems quite complex and must be clarified.

Regarding study limitations, since we only included sick leaves that had been officially reviewed, sickness absenteeism due to mental and behavioral disorders may be underestimated, since the since leaves < 5 days were not considered. In addition, occupational factors, such as working conditions, interpersonal conflicts, having a second job, length of service, etc., could have contributed to sickness absenteeism due to mental and behavioral disorders in this population. Nevertheless, the nature of the data did not allow such an analysis.

## CONCLUSIONS

The present study described the profile of sickness absenteeism due to mental and behavioral disorders among federal civil servants in Acre, Brazil, finding that these disorders, especially depressive episodes, caused the most lost workdays. These results indicate potential damage to civil servants and institutions, since these disorders are associated with higher levels of disability and a lower possibility of returning to work. In addition, the prevalence of recurrent leaves highlights the need for further research, since predictors of recidivism are still poorly understood.

It is also noteworthy that, although data on leaves due to mental disorders are scarce among Brazilian federal civil servants, they express a progressive increase in cases and an urgent need for policies to promote health and prevent mental disorders, minimizing their impact and promoting coping strategies at both the individual level and in the work environment. Thus, studies should assess the impact of conditions and the organization of work processes on the mental health of these workers.
